# Clinical Efficacy of Bolus of Six Drugs Including Rehmannia as an Adjunct to Metformin in the Treatment of Senile Type-2 Diabetes Mellitus and its Influence on Insulin Resistance, Inflammatory Factors and Blood Glucose-related Indicators

**DOI:** 10.12669/pjms.39.5.7262

**Published:** 2023

**Authors:** Ning Li, Yanru Yao, Erhui An

**Affiliations:** 1 Ning Li, Department of Internal Medicine, Baoding Hospital of Traditional Chinese Medicine, Baoding, Hebei, 071000, China; 2 Yanru Yao, Department of Internal Medicine, Baoding Hospital of Traditional Chinese Medicine, Baoding, Hebei, 071000, China; 3 Erhui An, Department of Internal Medicine, Baoding Hospital of Traditional Chinese Medicine, Baoding, Hebei, 071000, China

**Keywords:** Type-2 diabetes mellitus, Bolus of Six Drugs Including Rehmannia, Metformin, Insulin resistance, Inflammatory factors

## Abstract

**Objective:**

To investigate the clinical efficacy of Bolus of Six Drugs Including Rehmannia (Liuwei Dihuang pill) as an adjunct to metformin in the treatment of senile Type-2 diabetes mellitus and its influence on insulin resistance, inflammatory factors and blood glucose-related indexes.

**Methods:**

This is a Retrospective study. Eighty senile Type-2 diabetes mellitus admitted to Baoding Hospital of Traditional Chinese Medicine from January 2019 to December 2021 were enrolled and divided into two groups using the random number method. Patients in the control group were given oral metformin, while those in the observation group were treated with Bolus of six Drugs Including Rehmannia as an adjunct to metformin. The clinical efficacy, blood glucose-related indicators, insulin-related indicators, inflammatory factors-related indicators and adverse drug reactions were compared between the two groups.

**Results:**

The overall response in the observation group was higher than that in the control group(*P*<0.05). After treatment, the levels of FPG, 2hPG and HbA1 being more significantly lower in the observation group than that in the control group(*P*<0.05). Moreover, the levels of FINS, HOMA-IR and HOMA-IS were all significantly improved in the observation group than that the control group (*P*<0.05). HOMA-β levels in the observation group were significantly higher than those in the control group (*P*<0.05). The levels of TNF-α, IL-6 and IL-8 in the observation group were significantly lower than those in the control group (*P*<0.05).

**Conclusion:**

Bolus of six Drugs Including Rehmannia as an adjunct to metformin is a regimen with satisfactory safety profile for the treatment of senile Type-2 diabetes mellitus.

## INTRODUCTION

Diabetes mellitus is a clinical chronic metabolic disease caused by defects in the secretion and action of insulin, resulting in elevated blood glucose levels in the body.^1^ Diabetes usually manifests as two types, Type-1 and Type-2. Clinically, Type-2 diabetes is highly prevalent, accounting for 90-95% of all diabetes mellitus, mostly in the middle-aged and elderly population.^2^,^3^ If no effective control is targeted for increased blood glucose, complications such as diabetic nephropathy, peripheral neuropathy and coronary artery disease may occur secondary to diabetes mellitus.^4^

Therefore, early treatment and prevention of complications are crucial for the treatment of Type-2 diabetes mellitus (T2DM). Currently, hypoglycemic drugs are preferred as treatment for T2DM, with metformin being the most commonly used. It has been clinically proven to be clinically effective in lowering blood glucose and altering blood flow.^5^ According to traditional Chinese medicine, T2DM belongs to the category of “emaciation-thirst disease”^6^ which is mainly manifested as deficiency of the Kidney-Yin and deficiency of both Qi and Yin in patients. When treating this disease, priority should be given to adjusting Yin and Yang of the body and ameliorating the function of viscera.^7^

Bolus of six drugs including Rehmannia, as a traditional Chinese medicine, boasts various effects such as nourishing the liver and kidney, regulating the balance of Yin and Yang in the kidney, clearing the deficiency of fire, reducing blood sugar, and improving glucose tolerance. In this study, the effect of this regimen on clinical outcome, insulin resistance, glycemic control and serum inflammatory factors in elderly patients with T2DM was analyzed on the basis of the combined treatment of Bolus of Six Drugs Including Rehmannia and metformin.

## METHODS

This is a Retrospective study. Eighty senile Type-2 diabetes mellitus admitted to Baoding Hospital of Traditional Chinese Medicine from January 2019 to December 2021 were recruited and divided into two groups using the random number method: the observation group and the control group, with 40 cases in each group.

### Ethical Approval:

The study was approved by the Institutional Ethics Committee of Baoding Hospital of Traditional Chinese Medicine (No.:2022006; date: October 12, 2022), and written informed consent was obtained from all participants.

### Inclusion criteria:

- Patients who conformed to the Guideline for the Prevention and Treatment of Type-2 Diabetes Mellitus in China (2013 Edition)^8^;
- Patients with internal heat due to Yin deficiency diagnosed in Chinese medicine by the Guidelines for TCM Diabetes Prevention and Treatment: polyphagia, polydipsia, polyuria, bulimia, aversion to heat and preference for cold, dry and hard stools, deep-colored urine, reddened tongue and yellow tongue fur^9^;
- Patients aged ≥60 years;
- Patients with normal liver and kidney function;
- Patients who gave informed consent to this study and signed the consent Form.

### Exclusion criteria:

- Patients allergic to the study drug;
- Patients with cognitive impairment and difficult to actively cooperate with research;
- Patients with abnormal liver and kidney function and complicated with malignant tumors or severe infectious diseases.

The control group was given oral metformin at 0.5g/time, two times/d for the first time, after which the dose was adjusted according to the patient’s blood glucose level to a maximum of 1.0g/time, bid. In contrast, the observation group was given oral adjuvant therapy with Bolus of Six Drugs Including Rehmannia on top of the therapy in the control group, at eight capsules/time, tid. Patients in both groups were treated for 12 consecutive weeks.

### Observation indicators:

***Blood glucose-related indicators:*** The fasting plasma glucose (FPG), post-prandial two hour peripheral blood glucose (2hPG), and glycosylated hemoglobin A1c (HbA1c) levels were detected. *Clinical efficacy:* Markedly effective: symptoms of thirst disappeared after treatment, and FPG, 2hPG and HbA1c decreased to normal range; Effective: symptoms improved significantly after treatment, and FPG and 2hPG decreased by at least 20%; Ineffective: symptoms did not improve significantly or even worsened, and FPG, 2hPG and HbA1c did not reach the target or even increased. Overall response rate = markedly effective rate + effective rate.

***Insulin-related indicators:*** Radioimmunoassay was used to detect the fasting insulin level (FINS) of patients, and it was calculated that homeostasis model assessment - insulin resistance index (HOMA-IR)=(FPG×FINS)/22.5, homeostasis model assessment - β cell function index (HOMA-β)=20×FINS/FPG, and homeostasis model assessment - insulin sensitivity index (HOMA-IS)=20×FINS/(FPG-3.5).

***Inflammatory factor-related indicators:*** The tumor necrosis factor-α (TNF-α), interleukin-6 (IL-6) and interleukin-8 (IL-8) levels before and after treatment in both groups were detected. Furthermore, comparison of adverse drug reactions between the two groups was also made.

### Statistical Analysis:

All data in this study were statistically analyzed using SPSS22.0 software. Measurement data were expressed as mean ± standard deviation (*X̅*±*S*), and t test was used for comparison. Enumeration data was expressed as n (%), and c² test was used for comparison between the two groups. *P*<0.05 indicates a statistically significant difference.

## RESULTS

No statistically significant difference was observed between the two groups in terms of gender, age, weight and BMI (*P*>0.05), which was comparable. Table-I. After treatment, the overall response rate in the observation group was 95.00%, which was higher than 80.00% in the control group (*P*<0.05, Table-II).

**Table-I:** Comparison of general clinical data between two groups.

Group	n	Gender (cases)		Age (years)	Weight (Kg)	BMI (kg/m^2^)

		Male	Female			
Observation group	40	25	15	69.13±5.22	67.40±7.07	24.59±3.73
Control group	40	27	13	69.30±4.86	68.99±5.67	23.53±4.18
t/χ² value		0.220		0.155	1.109	1.197
*P* value		0.639		0.877	0.271	0.235

**Table-II:** Comparison of clinical efficacy between the two groups [n (%)].

Group	n	Markedly effective	Effective	Ineffective	Overall response rate (%)
Observation group	40	34 (85.00)	4 (10.00)	2 (5.00)	39 (95.00)
Control group	40	29 (72.50)	3 (7.50)	8 (20.00)	34 (80.00)
χ^2^ value					4.114
P value					0.043

After treatment, the levels of FPG, 2hPG and HbA1 in both groups were significantly lower than those before treatment (*P*<0.05), and the change was more pronounced in the observation group (*P*<0.05, Table-III).

**Table-III:** Comparison of blood glucose indicators between the two groups (*χ̅*±*S*).

Observation indicator	Observation point	Observation group	Control group	t	p
FPG (mmol/L)	Before treatment	14.33±1.86	14.15±2.49	0.356	0.723
	After treatment*	7.49±1.63	11.09±2.14	8.670	0.010
2hPG (mmol/L)	Before treatment	13.70±1.83	14.60±2.44	1.853	0.068
	After treatment*	8.80±1.51	10.02±2.24	2.822	0.006
HbA1c (%)	Before treatment	10.38±2.79	10.22±2.48	0.271	0.787
	After treatment*	6.61±1.89	8.09±2.55	2.959	0.004

***Note:***

* P<0.05 compared with the same group before treatment.

The levels of FINS, HOMA-IR and HOMA-IS were all significantly improved compared with those before treatment (*P*<0.05), and the improvement degree of the observation group was more obvious than that of the control group (*P*<0.05). The level of HOMA-β was higher in the observation group than in the control group (*P*<0.05, Table-IV).

**Table-IV:** Comparison of insulin-related indicators between the two groups (*χ̅*±*S*).

Observation indicator	Observation point	Observation group	Control group	t	p
FINS (mU/L)	Before treatment	13.68±2.41	13.74±2.98	0.103	0.918
	After treatment	8.45±1.81*	11.14±2.19*	6.008	0.000
HOMA-IR	Before treatment	8.84±2.43	8.82±2.91	0.035	0.972
	After treatment	2.88±1.08*	5.62±1.87*	8.033	0.000
HOMA-β	Before treatment	19.11±2.39	19.64±4.40	0.672	0.504
	After treatment	22.90±4.65*	20.31±3.49^Δ^	2.812	0.006
HOMA-IS	Before treatment	25.47±3.32	26.77±7.46	0.925	0.358
	After treatment	46.44±14.90*	30.64±6.91*	6.084	0.000

***Note:***

* P<0.05 compared with the same group before treatment;

Δ *P*>0.05 compared with the same group before treatment.

The levels of TNF-α, IL-6 and IL-8 were significantly lower in both groups compared with those before treatment (*P*<0.05), and the levels were more significantly reduced in the observation group than in the control group (*P*<0.05, Table-V). No statistically significant difference was observed in the incidence of adverse reactions between the two groups (*P*>0.05). Table-VI.

**Table-V:** Comparison of inflammatory factors between the two groups (*χ̅*±*S*).

Observation indicator	Observation point	Observation group	Control group	t	p
TNF-α (ng/L)	Before treatment	1.76±0.18	1.73±0.16	0.591	0.556
	After treatment*	1.10±0.10	1.28±0.07	9.063	0.000
IL-6 (ng/L)	Before treatment	112.66±7.68	112.26±6.09	0.257	0.798
	After treatment*	53.82±6.00	57.53±5.57	2.866	0.005
IL-8 (ng/L)	Before treatment	10.42±3.84	10.84±3.16	0.531	0.597
	After treatment*	5.51±2.45	6.81±2.76	2.227	0.029

***Note:***

* P<0.05 compared with the same group before treatment.

**Table-VI:** Comparison of adverse reactions between the two groups after treatment [n (%)].

Group	n	Drowsiness	Nausea	Anorexia	Incidence of adverse reactions
Observation group	40	2 (5.00%)	0 (0.00%)	1 (2.50%)	3 (7.50%)
Control group	40	2 (5.00%)	1 (2.50%)	1 (2.50%)	4 (10.0%)
c^2^					0.157
P					0.692

## DISCUSSION

The main pathogenesis of diabetes mellitus is insulin resistance and deficiency of insulin secretion.^10^,^11^ Currently, T2DM is treated clinically with a focus on ameliorating insulin function and promoting insulin secretion^12^, with hypoglycemic drugs being preferentially chosen to lower patients’ blood glucose. There are a variety of hypoglycemic drugs available clinically, among which metformin is widely used for the treatment of diabetes. With the progress of medical research, high attention has gradually been placed on the role of traditional Chinese medicine in the treatment of diabetes in clinical practice. In this study, from the perspective of improving clinical efficacy and reducing inflammatory response, it was found that Bolus of Six Drugs Including Rehmannia supplemented with metformin is a safe regimen for the treatment of Type-2 diabetes in the elderly.

According to traditional Chinese medicine, diabetes mellitus should be treated by nourishing the kidney and liver as well as nourishing yin and clearing heat.^13^ Bolus of Six Drugs Including Rehmannia is a classic Chinese herbal formula for nourishing the liver and kidney. In this formula, rehmannia is used to nourish the liver and kidneys, nourish Yin and tonify the kidneys; Fructus Corni nourishes the liver and kidneys and has the effect of reinforcing the kidney and controlling noctural emission; Chinese yam can tonify Yin and strengthen the spleen; Cortex Moutan clears deficiency heat; Radix Rehmanniae and Astragalus mongholicus nourish Yin and clear heat; peach kernel has the effect of activating blood circulation and resolving blood stasis; Ginger can lower the adverse flow of Qi, stop vomiting, enhance insulin release, and prevent the occurrence of diabetic complications; and licorice has the effect of reconciling various medicines. With the combined application of these drugs, the effect of resolving fatigue and eliminating turbidity, nourishing Yin and lowering fire can be achieved.^14^

In this study, FPG, 2hPG, HbAlc, FINS and HOMA-IR were significantly lower in the observation group than in the control group, while HOMA-IS was higher than in the control group, suggesting the satisfactory efficacy of Bolus of Six Drugs Including Rehmannia in regulating blood glucose levels and improving insulin resistance in patients with T2DM. This was consistent with the conclusion of Wang et al^15^, which further indicates that Bolus of Six Drugs Including Rehmannia combined with metformin is better than metformin alone in controlling blood glucose in patients with T2DM.

TNF-α, IL-6 and IL-8, as important inflammatory factors, are involved in the inflammatory response of the body.^16^ It has been shown in a study^17^ that the elevated levels of inflammatory factors such as TNF-α and IL-6 are vital signs for the development of diabetic nephropathy, and that the inflammatory response of the body can be reduced by applying traditional Chinese medicine to tonify the kidney and dredge collaterals. In this study, the levels of TNF-α, IL-6 and IL-8 in the observation group were significantly lower than those in the control group after treatment, which was consistent with the conclusions of previous studies^18^,^19^, demonstrating the efficacy of Bolus of Six Drugs Including Rehmannia in effectively inhibiting the inflammatory response of the organism and promoting its recovery.

Metformin has been clinically proven to be effective and safe by virtue of its clinical application for many years^20^, while no systematic study has been conducted on the mechanism of drug interaction between the combined application of Bolus of Six Drugs Including Rehmannia and metformin. The present study did not show any serious adverse reactions during treatment in the two groups. It showed the good clinical safety of Bolus of Six Drugs Including Rehmannia and metformin when applied in combination, which is consistent with the results of several clinical studies. The conclusion of this study provides a new clinical reference for the treatment of senile Type-2 diabetes mellitus with Bolus of Six Drugs Including Rehmannia combined with metformin.

### Limitations:

It includes fewer observation cases and the follow-up time was short. Another important limitation is that in order to find out the safety and efficacy of (Liuwei Dihuang pill) head to head comparison should be undertaken with metformin. In future we plan to include more cases and the follow-up time will be extended to further evaluate the clinical application value of Bolus of Six Drugs Including Rehmannia combined with metformin.

## CONCLUSION

Bolus of Six Drugs Including Rehmannia (Liuwei Dihuang pill) as an adjunct to metformin is a regimen with satisfactory safety profile for the treatment of senile Type-2 diabetes mellitus, boasting reduced insulin resistance in these patients, improved clinical outcomes and reduced inflammatory response of the body.

### Authors’ Contributions:

**NL:** Carried out the studies, participated in collecting data, drafted the manuscript, are responsible and accountable for the accuracy and integrity of the work.

**YY:** Performed the statistical analysis and participated in its design.

**EA:** Participated in acquisition, analysis, or interpretation of data and draft the manuscript.

All authors read and approved the final manuscript.
